# Efficacy of prebiotics and probiotics for functional dyspepsia: A systematic review and meta-analysis

**DOI:** 10.1097/MD.0000000000019107

**Published:** 2020-02-14

**Authors:** Jiaqi Zhang, Hao Meng Wu, Xue Wang, Jingyi Xie, Xia Li, Jinxin Ma, Fengyun Wang, Xudong Tang

**Affiliations:** a Department of Gastroenterology, Xiyuan Hospital of China Academy of Chinese Medical Sciences, Beijing; b Department of Gastroenterology, The Second Affiliated Hospital of Guangzhou University of Chinese Medicine, Guangzhou; c Experimental Research Center of China Academy of Chinese Medical Sciences; d Beijing University of Chinese Medicine; e China Academy of Chinese Medical Sciences, Beijing, China.

**Keywords:** functional dyspepsia, meta-analysis, prebiotics, probiotics, synbiotics

## Abstract

**Background:**

Functional dyspepsia (FD) is a functional gastrointestinal disorder. Evidence suggests that disturbance of the gastrointestinal microbiota may be implicated in FD. We performed a systematic review and meta-analysis to examine the efficacy of prebiotics and probiotics for FD.

**Methods:**

MEDLINE, EMBASE, and the Cochrane Controlled Trials Register were searched (through September 2018). Randomized controlled trials (RCTs) that recruited adults with FD and that compared prebiotics, probiotics, or synbiotics with placebo or no therapy were eligible. Eligibility assessment and data extraction were performed by two independent researchers. Dichotomous symptom data were pooled to obtain a relative risk (RR) with a 95% confidence interval (CI) of remaining symptomatic after therapy. Continuous data were pooled using a standardized or weighted mean difference with a 95% CI.

**Results:**

The search strategy identified 1062 citations. Five RCTs were eligible for inclusion. The RR of FD symptoms improving with probiotics or probiotics vs placebo was 1.15 (95% CI 1.01–1.30). Probiotics and prebiotics had beneficial effects on symptom scores of FD. Data for synbiotics in the context of FD were sparse, and no definite conclusions could be drawn.

**Ethics and dissemination:**

This study belongs to the category of systematic reviews, not clinical trials. Therefore, it does not require ethical approval. The results of this study will be published in influential international academic journals related to this topic.

**Conclusion:**

Probiotics and prebiotics seemed to be effective treatments for FD, although the individual species and strains that are the most beneficial remain unclear. Using only probiotics failed to improve the symptoms of FD. Further evidence is required before the role of probiotics, prebiotics, and synbiotics in FD can be fully understood.

## Abstract 2

Supplemental Digital Content is available in the text

## 1 Introduction

As a chronic disorder of the gastroduodenal region, functional dyspepsia (FD) is a common disease of the digestive system.^[1]^ According to the Rome IV criteria, FD is defined as the presence of at least one of the following symptoms: postprandial fullness, early satiation, epigastric pain, or burning, a lack of evidence of structural disease to explain the symptoms fulfilling the time criteria of the last 3 months with symptom onset at least 6 months before diagnosis and a frequency of at least 3 days per week.^[2,3]^ There are no organic diseases evidenced by an upper gastrointestinal (GI) endoscopy that can explain the symptoms. The prevalence of FD varies widely across the globe, with an incidence of 10% to 40% in Western countries and 5% to 30% in Asian countries, independent of their definition of FD.^[4,5]^ Up to 40% of persons who have FD consult a physician due to unbearable symptoms.^[6]^ Because of consultations for symptoms, physical examinations, medications, and sickness-related absences from work, FD has a significant impact on personal quality of life, economy, health services, and society.^[7]^ Functional dyspepsia is considered a multifactorial disorder in which a number of putative pathophysiological mechanisms, including altered GI motility, visceral hypersensitivity, dysregulation of the gut–brain axis, psychological disturbances, low-grade inflammation, and immune system dysfunction, have been proposed.^[8]^ Evidence suggests that intestinal flora imbalance is involved in the development of FD.^[9–11]^

Because the exact cause of FD is still ambiguous, there is no definitive treatment that is beneficial to all individuals. Recently, research has shown that probiotics may be beneficial for patients with FD.^[12]^ Probiotics are active microorganisms that are beneficial to the host and have been reported to be effective in the treatment of functional GI diseases, especially irritable bowel syndrome (IBS).^[13,14]^ To date, whether probiotics can improve FD remains controversial. The beneficial effect of probiotics on IBS is believed to result in reduced low-grade inflammation and improved mucosal permeability by reducing abnormalities of the intestinal flora.^[15]^ Moreover, due to inflammation and mucosal damage, the duodenum has recently received attention as an organ involved in the pathogenesis of FD,^[16]^ where a large number of intestinal flora colonize. Thus, modulating the gut microbiota, as a means of improving symptoms, may be a beneficial treatment option.

Recently, to assess the efficacy and safety of probiotics or prebiotics in individuals with FD, several randomized controlled trials (RCTs) have been conducted^[12,17,18]^; however, some studies have included very few patients, and the results are very contradictory. Therefore, the role of probiotics or prebiotics in the management of FD is currently unclear. To address this uncertainty, we conducted a systematic review and meta-analysis of RCTs to assess the efficacy and safety of probiotics and prebiotics in patients with FD.

## 2 Methods

### 2.1 Search strategy and study selection

A search of the medical literature was conducted using MEDLINE (1946 to September 30, 2018), EMBASE, EMBASE Classic (1947 to September 30, 2018) and the Cochrane central register of controlled trials. Randomized placebo-controlled trials examining the effect of prebiotics, probiotics, and synbiotics in adult patients (over the age of 16 years) with FD were eligible for inclusion (see Box 1, Supplemental Content, which illustrates the eligibility criteria). The duration of therapy had to be at least 7 days. The diagnosis of FD could be based on either a physician's opinion or symptom-based diagnostic criteria, with a negative upper GI endoscopy excluding an organic cause of dyspepsia. Subjects were required to be followed for at least 1 week, and studies had to report a global assessment of FD symptom cure or improvement after the completion of therapy, preferably as reported by the patient, but if this was not recorded, then as documented by the investigator. When studies did not report these types of data but were otherwise eligible for inclusion in the systematic review, we attempted to contact the original investigators to obtain dichotomous data.

Studies on FD were identified with the term dyspepsia (as a medical subject heading [MeSH] and a free text terms), and dyspep$, satiety, epigastric adj5 pain, upper GI symptom$, or upper GI symptom$ (as free text terms). These terms were combined using the set operator AND with studies identified with the following terms: *Saccharomyces*, *Lactobacillus*, *Bifidobacterium*, *Escherichia coli*, probiotics, synbiotics, or prebiotics (both as MeSH and free text terms).

There were no language restrictions, and abstracts of the papers identified by the initial search were evaluated by two reviewers for appropriateness in relation to the study topic. All potentially relevant papers were obtained and evaluated in detail. Foreign language papers were translated when necessary. The bibliographies of all identified relevant studies were used to perform a recursive search of the literature. Articles were assessed independently by two reviewers using predesigned eligibility forms, according to the prospectively defined eligibility criteria. Any disagreement between investigators was resolved by consensus.

### 2.2 Outcome assessment

The primary outcomes assessed were the effects of prebiotics, probiotics, or synbiotic drugs compared with those of placebo on global FD symptoms after cessation of therapy. Secondary outcomes were adverse events as a result of treatment. The primary outcome and the secondary outcome were both categorical variables.

### 2.3 Data extraction

All data were extracted independently by two reviewers using a Microsoft Excel spreadsheet (2010 Edition; Microsoft Corp, Redmond, WA). All data extracted were then checked by a third reviewer. In addition, the following clinical data were extracted for each trial: setting (primary, secondary, or tertiary care-based), number of centers, country of origin, dose and duration of treatment, total number of adverse events reported, criteria used to define FD, primary outcome measure used to define symptom improvement or cure following treatment, duration of treatment, duration of follow-up, and proportion of female patients. Data were extracted based on intention-to-treat analyses, where all drop-outs were assumed to be treatment failures when ever trial reporting allowed this (see Box 2, Supplemental Content, which illustrates the data extraction methodology.)

### 2.4 Assessment of risk of bias

This assessment was performed independently by two investigators, with disagreements resolved by consensus. The risk of bias was assessed as described in the Cochrane Handbook^[19]^ by recording the method used to generate the randomization schedule and conceal allocation, whether blinding was implemented for participants, personnel, and outcome assessment, what proportion of subjects completed follow-up, and whether there was evidence of selective reporting of outcomes.

### 2.5 Data synthesis and statistical analysis

*I*^2^ statistics were used to evaluate between-study heterogeneities. Random-effect models (DerSimonian–Laird method) were used if *I*^2^ exceeded 50%. Otherwise, meta-analyses were conducted with fixed-effect models (Mantel–Haenszel method).^[20]^ The impacts of different interventions were expressed as a relative risk (RR) of global FD symptoms not improving with probiotics and prebiotics compared with placebo, with 95% CIs. Adverse event data were also summarized with RRs.

Heterogeneity was assessed using the *I*^2^ statistic, with a cut-off of ≥50%. A χ^2^ test with a *P* < .10 used to define a significant degree of heterogeneity.^[21]^ When the degree of statistical heterogeneity was greater than the between-trial results in this meta-analysis, possible explanations were investigated using subgroup analyses according to type of psychotropic drug used, trial setting, criteria used to define FD. These were exploratory analyses only and may explain some of the observed variability, but the results should be interpreted with caution.

Review Manager V.5.3 was used to generate forest plots of pooled RRs for primary and secondary outcomes with 95% CIs, as well as funnel plots. The latter were assessed for evidence of asymmetry, and therefore possible publication bias or other small study effects were assessed using the Egger test^[22]^ if there were sufficient (10 or more) eligible studies included in the meta-analysis, which is in line with current recommendations.^[23]^

## 3 Results

**Figure 1:**
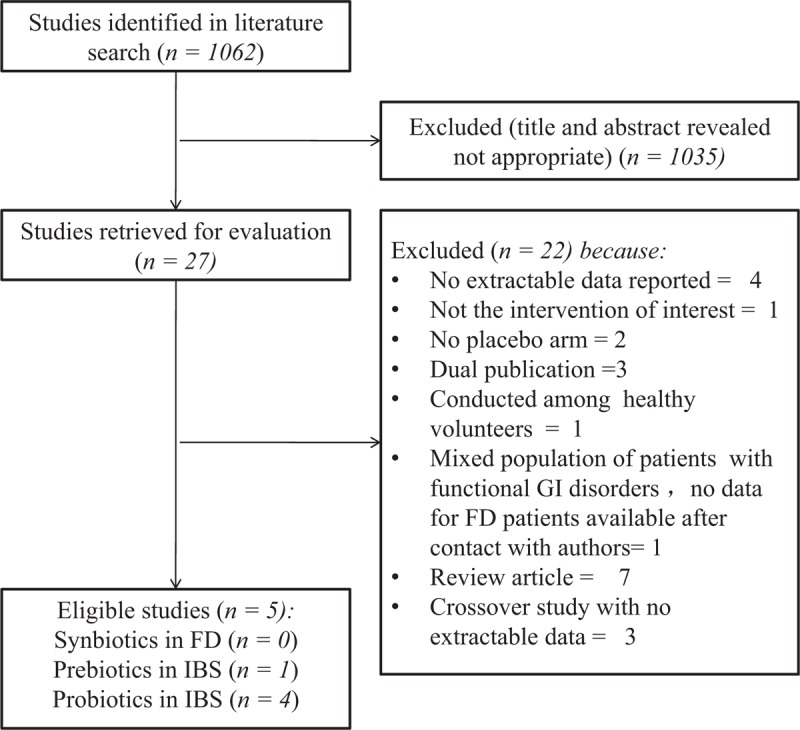
Flow diagram of assessment of studies identified in the updated systematic review and meta-analysis.

The search strategy identified a total of 1062 citations, of which 27 published articles appeared to be relevant and were retrieved for further assessment. Of these 27 articles, 22^[18,24–44]^ were excluded for various reasons, leaving only 5 eligible studies (Fig. 1). Agreement between reviewers for assessment of trial eligibility was good (κstatistic = 0.91). We successfully contacted original investigators to seek clarification on study methodology and hence reduce the risk of bias, and we obtained supplementary dichotomous data for eight trials.^[17,45–48]^ We identified 4 RCTs of probiotics for FD^[17,45–47]^ and one study of prebiotics.^[48]^

The 5 RCTs of probiotic and prebiotic drugs for FD involved 409 patients. The proportion of female patients recruited for the trials ranged from 51.0% to 80%. All five trials were at a low risk of bias. Four trials used a combination of probiotics, included *Lactobacillus*, *Bifidobacterium*, *Streptococcus*, *Bacillus subtilis*, and *Bacillus lichenformis*. The detailed characteristics of the individual RCTs are provided in Table 1.

**Table 1:**
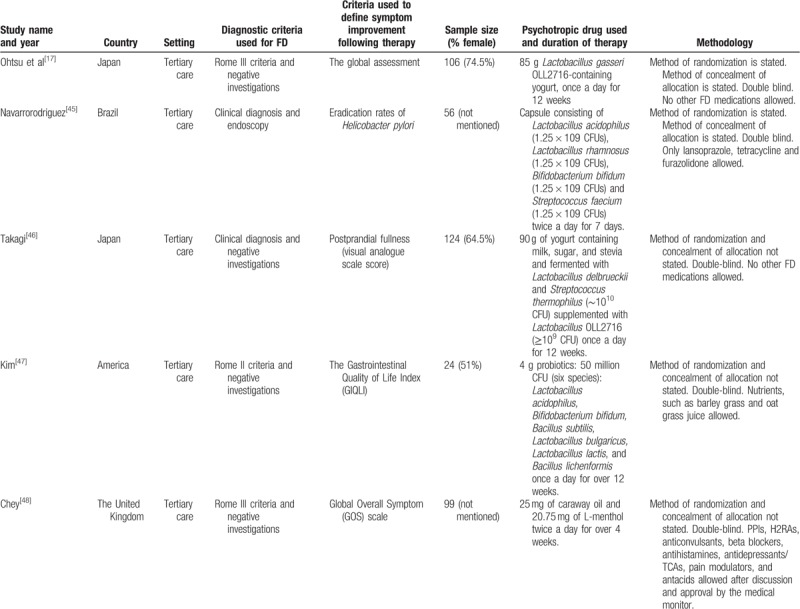
Characteristics of randomized controlled trials of probiotics vs placebo for functional dyspepsia.

### 3.1 Efficacy of probiotic and prebiotic drugs for the treatment of FD

**Figure 2:**
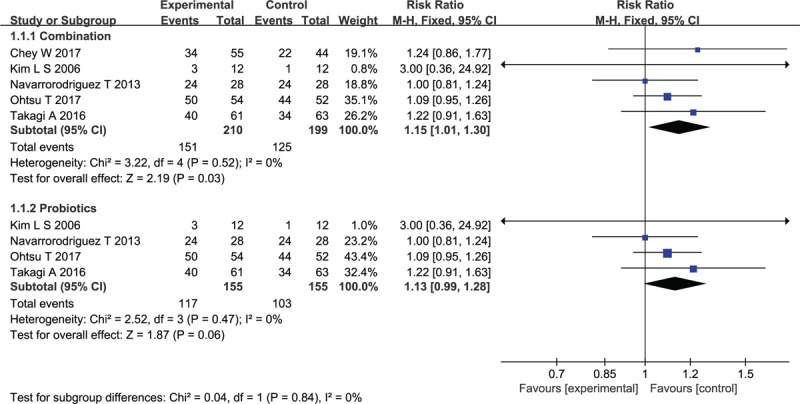
Forest plot of efficacy of probiotics and prebiotics drugs versus placebo in randomized controlled trials in functional dyspepsia.

**Figure 3:**
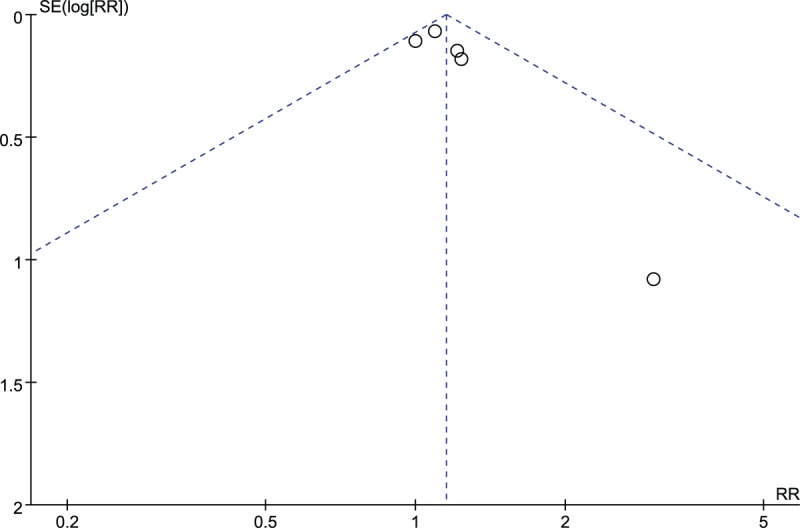
Funnel plot of publication bias of the included studies.

In total, there were 409 patients, 210 of whom received active therapy and 199 received placebos. Overall, 59 (28.1%) of 210 patients assigned to probiotic or prebiotic groups reported improved FD symptoms following therapy, compared with 74 (37.2%) of the 199 patients allocated to the placebo group. The RR of FD symptom improvement after treatment with probiotics or prebiotics vs placebo was 1.15 (95% CI 1.01–1.30), with a statistically low degree of heterogeneity detected between studies (*I*^2^ = 0%, *P* = .52; Fig. 2). The studies were more evenly distributed on both sides of the combined effect, indicating that publication bias was small, but most of the studies were concentrated in the upper part of the funnel plot, so there was a risk of missing small sample studies (Fig. 3). Probiotics were assessed in 4 RCTs,^[46–49]^ comprising 310 patients, with no demonstrated effect on symptom improvement (RR = 1.13; 95% CI 0.99–1.28; Fig. 2) and low degree heterogeneity between studies (*I*^2^ = 0%, *P* = .67).

### 3.2 Adverse events associated with probiotics and prebiotics drugs

**Figure 4:**
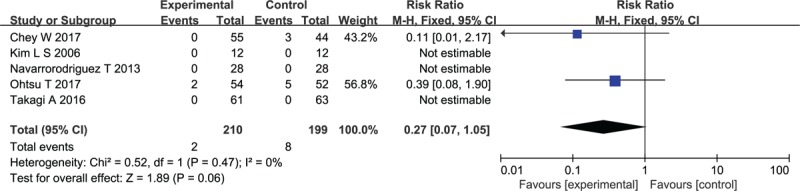
Forest plot of safety profile and adverse events of the included studies.

Data concerning total numbers of adverse events were available for 2 of the trials. A total of 2 (1.83%) of the 109 patients assigned to the probiotics group experienced an adverse event, compared with 8 (8.3%) of the 96 patients allocated to the placebo group. When data were pooled, there was no difference in adverse events in the probiotic group vs the placebo group (RR of experiencing any adverse event = 0.27; 95% CI 0.07–1.05) with no heterogeneity between results (*I*^2^ = 0%, *P* = .47) (Fig. 4).

## 4 Discussion

This meta-analysis demonstrated that probiotics and prebiotics appear to be a beneficial therapy for FD. However, we have no evidence to support that probiotics are effective for FD treatment. However, in regard to improving FD symptoms, there was a trend toward a beneficial effect of probiotics, although it remains unclear which strain or species may be beneficial. Although adverse events are rare, they were more common in the placebo group. Only one clinical study showed the efficacy of prebiotics for FD, which appeared to be of no benefit. Unfortunately, there was no synbiotic trial that met the inclusion criteria. We also contacted researchers of studies that may have been eligible for inclusion to obtain supplemental bibliographic data on treatment outcomes of unrecognized treatments, for symptoms and adverse events in the original publication, or to clarify research methods to minimize the risk of inclusion bias among the RCTs. However, RCTs using probiotics as a treatment for FD are rare. In the end, we only included 5 studies that provided us with data from 409 FD patients treated with probiotics or prebiotics versus placebo. We performed subgroup analyses to explore the effect of probiotics on FD, and based on the individual treatment used, study setting, criteria used to define FD, and risk of bias of included studies, we assessed the treatment effect. Finally, to maximize the data available for synthesis, we extracted and merged adverse events. In the included studies, *Lactobacillus* was the most commonly used for the treatment of FD, followed by *Bifidobacterium*, *Streptococcus*, and *Bacillus*. According to the RR, the combination of multiple probiotics to treat FD exerted a more obvious effect, suggesting that we can consider the combination of probiotics and prebiotics in the treatment of FD to restore the disordered GI microecology and improve FD symptoms.

There were limitations to this meta-analysis, some of which arise from the heterogeneity and sample sizes of the studies available for inclusion. Due to a lack of reporting of the methods used to generate the randomization schedule and conceal allocation, three included trials had an unclear risk of bias,^[48–50]^ which could lead to overestimation of treatment outcomes. Subjective, dichotomous results, rather than mechanical endpoints, may have led to the higher placebo response rates in all included trials, which is a common problem in FD clinical trials. It should also be noted that some studies that included individuals infected with *Helicobacter pylori* may limit the application of our findings to FD patients. In addition, for any of the RCTs we identified, the longest duration of treatment was 12 weeks, which means that the long-term efficacy of probiotics or prebiotics for FD is unclear. We attempted to reveal which species and strains of probiotics were effective, but the limited number of trials in these subgroup analyses meant that we may not have enough power to detect any meaningful difference in efficacy. However, a single strain of probiotics may have different effects, and aggregating all studies from a particular species may obscure the beneficial effects of a single strain of that species, although if more assessable studies examine each of these individual strains, we may be more able to judge their efficacy and compare the efficacy between strains.

Our study is the first meta-analysis to assemble all available data for the use of prebiotics, probiotics, and synbiotics for FD, although only 5 studies were included. Several studies have shown that probiotics and prebiotics are beneficial for functional GI diseases,^[49,50]^ but these studies have mainly focused on IBS.^[51,52]^ Probiotics are defined by the Food Agricultural Organization and the World Health Organization Expert Consultation on Evaluation of Health and Nutritional Properties of Probiotics. The definition is “Live microorganisms which, when administered in adequate amounts, confer a health benefit on the host.” A prebiotic is “anon digestible food ingredient that beneficially affects the host by selectively stimulating the growth and/or the activity of one or a limited number of bacteria in the colon,” thereby increasing the body's natural resistance to invading pathogens.^[53]^ The exact mechanism by which prebiotics are beneficial to host health needs further research to confirm. The increase in the number of beneficial bacteria and the fermentation of the prebiotics by the intestinal flora are the main factors affecting the health of the host's digestive tract. In addition, the intestinal flora produces SCFAs via the fermentation of prebiotics, mainly through the metabolism of butyric acid, acetic acid and propionic acid, to supply energy to the intestinal wall cells. Synbiotics are mixed products of probiotics and prebiotics or probiotics or prebiotics supplemented with vitamins and trace elements. Synbiotics cannot only exert the physiological bacterial activity of probiotics but also selectively increase the number of bacteria, making the probiotics more effective and lasting.^[54]^ A possible mechanism by which probiotics, prebiotics, and synbiotics improve GI disease by inhibiting pathogenic bacteria in intestinal epithelial cells, strengthening the barrier function of the intestinal epithelium, acidifying the colon, inhibiting the growth of pathogens, regulating immunity, inhibiting visceral hypersensitivity, changing mucosal stress response, and improving intestinal motor function.^[55]^ Muneki Igarashi^[19]^ posited that probiotics are effective for the treatment of FD by reducing the abundance of *Escherichia*/*Shigella*, a major source of toxic lipopolysaccharides, in the upper GI tract as well as restoring the change in the gastric microbiota.

There is still little evidence of the efficacy of prebiotics or synbiotics for FD, and further research is needed to determine its benefits. The mechanism of action of individual probiotics, such as *Bifidobacterium lactis*, in improving symptoms of FD remains speculative. Previous studies have shown that probiotics reduced visceral hypersensitivity by regulating the expression of pain receptors in the gut.^[56]^ Another study showed that *Lactobacillus paracasei* can improve intestinal motility by reducing glycogen synthesis, promoting the degradation of blood lipids, interfering with energy metabolism, and normalizing the smooth muscle function of the GI tract.^[57]^ Recent studies have shown that low-grade inflammation and increased duodenal mucosal permeability are important mechanisms of FD.^[58]^ The abundance of bacteria increases gradually from the stomach, duodenum to the small intestine, and probiotics are thought to improve mucosal permeability by improving abnormalities in the gut microbiota or to produce short-chain fatty acids via the fermentation of intestinal contents. Probiotics can directly participate, without the microbiota, in the amelioration of enhanced permeability.^[14,15,59]^ Moreover, research has shown that mucosal barrier function is improved by short-term active lactic acid bacteria treatment.^[60]^ However, further research is required to identify species and strains of probiotics that are consistently beneficial to uncover the mechanism for improving symptoms of FD and to elucidate how these benefits are achieved.

In summary, this meta-analysis has demonstrated little evidence for the use of prebiotics or synbiotics for FD. Using only probiotics failed to improve the symptoms of FD. Combinations of probiotics and prebiotics appeared to have the most evidence supporting their use, but more RCTs are needed before their true efficacy in treating this condition is known.

## Author contributions

**Data curation:** Xia Li, Jinxin Ma.

**Funding acquisition:** Jiaqi Zhang.

**Investigation:** Haomeng Wu.

**Methodology:** Haomeng Wu, Jingyi Xie, Jinxin Ma.

**Software:** Xue Wang, Jingyi Xie, Xia Li.

**Supervision:** Fengyun Wang.

**Writing – original draft:** Jiaqi Zhang, Xue Wang, Xia Li.

**Writing – review & editing:** Jiaqi Zhang, Xue Wang, Fengyun Wang, Xudong Tang.

## Supplementary Material

Supplemental Digital Content

## Supplementary Material

Supplemental Digital Content
