# Supplementary material for: Efficacy of prebiotics and probiotics for functional dyspepsia: A systematic review and meta-analysis
Source: Medicine (Baltimore). 2020 Feb 14;99(7):e19107. doi: 10.1097/MD.0000000000019107 (PMC7035106; doi:10.1097/MD.0000000000019107)
Supplement: Supplemental Digital Content [file medi-99-e19107-s001.docx]

**Box 1 Eligibility criteria**

| - Randomized controlled trials |
| --- |
| - Adults (participants aged >16 years) |
| - Diagnosis of functional dyspepsia based on either a   clinician’s opinion, or meeting specific diagnostic criteria*,   - Compared prebiotics, probiotics, or synbiotics with placebo |
| - Minimum duration of therapy 7 days. - Minimum duration of follow-up 7 days. - Dichotomous assessment of response to therapy in terms of   effect on global functional dyspepsia symptoms following therapy at study end  * Rome I, II,III or IV criteria or clinical diagnosis |
