# Supplementary material for: Efficacy of prebiotics and probiotics for functional dyspepsia: A systematic review and meta-analysis
Source: Medicine (Baltimore). 2020 Feb 14;99(7):e19107. doi: 10.1097/MD.0000000000019107 (PMC7035106; doi:10.1097/MD.0000000000019107)
Supplement: Supplemental Digital Content [file medi-99-e19107-s002.docx]

**Box 2.Data extraction methodology**

| - **Outcome of interest**: improvement in global functional dyspepsia symptoms. |
| --- |
| - **Reporting of outcomes**: patient-reported preferable, if not available then investigator-reported. |
| - **Time of assessment**: at last point of follow-up while still on therapy. |
| - **Denominator used**: true intention-to-treat analysis, if not available then all evaluable patients. Minimum duration of follow-up 7 days. - **Cut-off used for dichotomization:** any improvement in global functional dyspepsia symptoms or abdominal pain for Likert-type scales, investigator-defined improvement for continuous scales. |
